# Benefit finding in response to general life stress: measurement and correlates

**DOI:** 10.1080/21642850.2014.889570

**Published:** 2014-03-06

**Authors:** Tony Cassidy, Marian McLaughlin, Melanie Giles

**Affiliations:** ^a^ School of Psychology, University of Ulster, Cromore Road, Coleraine, Northern Ireland, BT52 1SA, UK

**Keywords:** benefit finding, psychological capital, social support, preventive interventions

## Abstract

Benefit finding herein defined as “the process of deriving positive growth from adversity” has become a key construct in the evolution of positive psychology, and research suggests that it may provide the basis for a resource model of stress and coping. However, measures of benefit finding have tended to be domain specific. The current study focused on developing a more generic multidimensional measure of benefit finding. A measure of benefit finding was developed and tested in 855 students (574 females and 281 males) aged between 18 and 40 years. A 28-item scale with six dimensions was produced and Confirmatory Factor Analysis (CFA) confirmed the scale structure. The model proposed that psychological and social resources would mediate the relationship between experienced stressors and benefit finding. Structural equation modelling with Analysis of Moment Structures (AMOS) shows that the model is a good fit for the data and psychological and social resources partially mediated the relationship. It is argued that psychological and social resources enable benefit finding in relation to life stress and provide a focus for the development of preventive interventions to improve positive health.

## Introduction

Around the turn of the century something akin to a paradigm shift occurred in the stress and coping literature with the evolution of a positive psychology approach based on constructs such as adversarial growth and benefit finding (Tennen & Affleck, 2002). This shift had been building for more than 20 years, largely from the work on post-traumatic growth (Linley & Joseph, 2004). There followed a growth in the research literature on benefit finding in chronic illness and disability (Helgeson, Reynolds, & Tomich, 2006), and more recently among family care givers (Cassidy, 2012; Cassidy, Giles, & McLaughlin, 2013). Helgeson et al. (2006) identified 235 studies, over half of which had been carried out between 2001 and 2006, and covered a range of chronic illnesses, war, sexual assault, disasters, and having a chronically ill child. Their meta-analysis concluded that benefit finding was related to lower levels of depression and more positive well-being, but was also correlated with more intrusive thoughts and was not correlated with self-rated physical health (Helgeson et al., 2006, p. 810). With well-established measures, the correlation between benefit finding and self-rated health reached significance, and as time from trauma onset increased the relationship with intrusive thoughts decreased (Helgeson et al., 2006, p. 809). The latter might suggest that emotional processing was taking place closer to the event and might ultimately lead to positive outcomes. Individual studies, however, have shown clear links between benefit finding and health. One of the first in the area was conducted by Affleck, Tennen, Croog, and Levine (1987), with 287 men who had recently experienced their first heart attack. Over 50% of men reported benefits and those who did were significantly less likely to have a subsequent heart attack and exhibited lower morbidity rates 8 years later, controlling for age, socio-economic status, and disease severity. Bower, Kemeny, Taylor, and Fahey (1998) showed benefit finding to be linked to less rapid decline in health and even reduced mortality in a sample of AIDS sufferers. Similar physiological benefits were shown in cancer patients (McGregor & Antoni, 2009; Stanton et al., 2002). However, the mechanisms linking benefit finding and health are still unclear (Bower, Moskowitz, & Epel, 2009) and the explication of these links may be enhanced by better measures. Bower et al. (2009) propose what could be described as a resource model of stress to explain the pathways from benefit finding to health, turning the traditional deficit model of stress (Lazarus & Folkman, 1984) on its head. They propose that benefit finding leads to increased psychological and social resources, through improving coping strategies, thereby making the individual more resilient and less responsive to stressful demands. However, as Bower et al. (2009) admit, “few studies have investigated whether benefit finding is associated with resource enhancement” (p. 340) which is a central tenet of their proposed model. This presupposes that benefit finding comes first, and while it is likely that reciprocal relations of causality exist, there is some evidence to suggest that positive psychological and social resources enable benefit finding to develop, or at least enhance its development (Cassidy, 2012; Cassidy et al., 2013). The latter would suggest that psychological and social resources mediate the relationship between stressors and benefit finding.

Helgeson et al.’s (2006) review includes research on growth (posttraumatic growth and stress-related growth) and they define benefit finding as “the positive effects that result from a traumatic event” (p. 797). While this breadth of coverage and definition suffice for their review, there are some issues that need to be unpacked. Firstly, they link benefit finding to traumatic events which begs the question regarding growth as a consequence of general life stress. The other issue is that as the literature has grown and measures have been developed it would appear that most researchers view benefit finding as a multidimensional construct which describes the processes involved in growth or benefit finding rather than just its existence (Tomich & Helgeson, 2004). These authors identified 6 factors, *acceptance*, *empathy*, *appreciation, family domain*, *positive self-view*, and *reprioritisation*. It seems more appropriate then to define benefit finding as “the process of growth”, and to allow the possibility that it may occur in more everyday setting as well as in response to trauma. In addition, past research has largely focused on demonstrating the existence of benefit finding following trauma and its relationship with health outcomes, with little or no research on factors that might contribute to or mediate benefit finding (Bower et al., 2009). In order to take forward a multidimensional model of benefit finding in the field of general life stress as opposed to chronic illness or trauma, there is a need for an appropriate measure. Only one multidimensional measure is to be found in the literature, the *Perceived Benefit Scale* (McMillen & Fisher, 1998) which is a 27-item scale measuring 8 factors, *lifestyle changes, material gain, increases in self-efficacy, family closeness, community closeness, faith in people, compassion,* and *spirituality.* Descriptions of these dimensions seem to indicate some overlap and commonality with the Tomich and Helgeson's (2004) 6-factor *Benefit Finding Scale.* Given the commonality and also the potential differences, it seems logical to test for this in a single sample which is a focus of the current study.

The only other potential measures are the Post-traumatic Growth Inventory (PTGI) (Tedeschi & Calhoun, 1996) and the Stress-Related Growth Scale (SRGS) (Park, Cohen, & Murch, 1996) often used to measure post-traumatic growth. Both scales have been widely used but there are a number of concerns regarding their validity (Bitsch, Elklit, Christiansen, 2011; Moore, Varra, Michael, & Simpson, 2010). Bitsch et al. argue that neither scale adequately defines growth or trauma and that it is unclear if the two scales really measure growth as opposed to coping attempts, positive reframing, or simply adaptation. This is borne out by a visual analysis of items in these scales. For example, items such as “I learned that it's OK to ask others for help” or “I learned to communicate more honestly with others” do not appear to have face validity.

The dimensionality of both measures is also questioned. The SRGS was originally used as a unidimensional measure but a number of studies have suggested that it is multidimensional (Armeli, Gunthert, & Cohen, 2001; Roesch, Rowley, & Vaughn, 2010). Interestingly, Park et al. (1996) developed the scale as a 3-factor model but analysis failed to replicate this and produced a single dimension. Armeli et al. (2001) produced a 7-factor solution and Roesch et al. (2010) were able to demonstrate 1, 3, and 7 factors but found the internal consistency of some factors to be suspect.

In order to explore the relationship between benefit finding and building positive health, we can draw on developments in positive psychology, for example, the work on psychological capital (Luthans, Youssef, & Avolio, 2007b). Luthans et al. (2007b) define psychological capital as “an individual's positive psychological state of development” (p. 3), and operationalise it as a composite of optimism, hope, self-efficacy, and resilience. However, while these are well-established constructs the overall conceptualisation seems to be missing a motivational element and two other constructs would seem to have potential in adding to the explanatory power. One is the long-established construct of self-determination (Deci & Ryan, 1985, 2000) and the other a more recent construct of life engagement (Harter, Schmidt, & Hayes, 2002; Seligman, 2011). This study adds these constructs and tests their fit in a model of psychological capital.

The other area of resource in regard to life stress is social support which is very widely established as a key factor in mediating and moderating stress and is linked both directly and indirectly to health (Cohen, 2004; Cohen, Gottlieb, & Underwood, 2000; Taylor, 2011). Traditionally research has focused on social support as a buffer to stress, or on the absence of support as a factor in illness and mortality; less effort has been devoted to exploring the positive role of support in building resilience and positive health. Most studies that claim positive impacts of social support measure it in terms of reduced negative consequences (Cohen, 2004). In this review, Cohen (2004) talks about the mechanisms whereby social support improves health by (1) improving coping resources and (2) reducing physiological processes that impact on ill health. In essence this supports the argument that social support is still generally viewed as a protective mechanism rather than a means of promoting positive health. Even interventions using social support tend to evaluate effectiveness in terms of reduced negative outcomes rather than increases in positive health and well-being (Hogana, Lindena, & Najarian, 2002; Small, Taft, & Brown, 2011). Some recent research suggests social support is linked to benefit finding (Luszczynska, Mohamed, & Schwarzer 2007) taking forward the resource building approach to social support. In psychological research, a distinction is made between received or actual social support (measured in terms of social connectedness) and perceived social support with studies generally showing only small correlations between the two (Barrera, 1986; Haber, Cohen, Lucas, & Baltes, 2007; Schwarzer & Leppin, 1991). Perceived support is generally seen as an individual difference variable (Sarason, Sarason, & Shearin, 1986). Perceived support tends to have the larger impact on outcomes, and in some studies it is only perceived support which has an impact (Taylor, 2011). Substantive conclusions that can be drawn from the social support literature are: (1) perceived support does not occur in the absence of actual support, (2) perceived support has the larger and more consistent impact, and (3) studies which only measure actual support produced equivocal findings (Cassidy, 2011, p. 15; Haber, et al., 2007). In this study, perceived social support is defined in terms of support from family and friends.

Research on benefit finding has traditionally focused on trauma or chronic illness. However, people encounter adversity in the events and hassles of everyday life as has been shown throughout the vast stress literature (Lazarus, 1993), which begs the question whether benefit finding can occur in such settings. Studies which have looked at benefit finding in chronically ill participants or survivors of trauma have generally shown a direct relationship between the level of trauma or illness and benefit finding (Helgeson et al., 2006). However, research on benefit finding among care givers has produced some evidence of an inverse relationship between the burden of care and benefit finding (Cassidy, 2012; Cassidy et al., 2013). This emerging evidence suggests that something different may be happening with care givers as opposed to survivors of trauma or chronic illness. An essential difference is that traumatic events and chronic illness are life threatening while care givers experience excessive demands but their life is not directly threatened. Where the burden of care giving is seen as unfair or developmentally inappropriate (as with young care givers), the care giver may feel overwhelmed and threatened rather than challenged by their load.

There have been a number of studies looking at stress-related growth in non-traumatised or chronically ill groups (Dolbier, Smith Jaggers, & Steinghardt, 2009). These studies use either the SRGS (Park et al., 1996) or the PTGI (Tedeschi & Calhoun 1996) which has recently been questioned in the literature. This raises questions about previous research using the measures and may partially explain the inconsistency in findings.

### Rationale

Given that there is substantial evidence that benefit finding is linked to improved health following adversity and that the pathways whereby this occurs are still unclear but are likely to conform to a resource model of stress (Bower et al., 2009), it is argued that a general measure of benefit finding that can be used outside of the areas of chronic illness, disability, and severe trauma, has potential utility. In addition, the lack of evidence on the link between benefit finding and resource building provides a gap that can begin to be addressed with such a measure. The current study aimed to develop such a measure and test a model whereby psychological and social resources mediate the relationship between stressors and benefit finding.

## Method

### Participants

A survey using questionnaire data collection was administered to a convenience sample of 855 undergraduate students, 574 females, and 281 males aged between 18 and 40 with a mean age of 23.4 (sd = 5.7).

### Measures

#### Benefit finding

Part of the focus of this study was to develop a benefit finding measure for general use drawing on two existing measures and a number of new items. The existing scales were selected because they were both multidimensional and covered the range of dimensions discussed in the literature. The first of these is the *Benefit Finding Scale* (Tomich & Helgeson, 2004) which is a 17-item scale developed through various stages from an original 30 items (Tomich & Helgeson, 2004). They identified 6 factors from the scale, *acceptance*, *empathy*, *appreciation, family domain*, *positive self-view*, and *reprioritisation*.

The second measure was the *Perceived Benefit Scale* (McMillen & Fisher, 1998) which is a 30-item scale measuring 8 factors, *lifestyle changes, material gain, increases in self-efficacy, family closeness, community closeness, faith in people, compassion,* and *spirituality*.

There appears to be some overlap between the scales and between them they appear to cover the domain of benefit finding pretty comprehensively. For example, the following item from the Tomich and Helgeson scale “Having had breast cancer, has helped me become a stronger person, more able to cope effectively with future life challenges”, compares with “this event made me a stronger person,” and “I am a more effective person because I went through this event”, from the McMillen and Fisher scale. On the other hand, the former scale has an item, “has led me to be more accepting of things” while the latter scale has no comparable item. The major difference between the two scales is that the Tomich and Helgeson scale focuses on a target population who have experienced a specific illness while the McMillen and Fisher scale asks participants to state how they generally responded to the most negative event experienced in the past five years. Both scales used a 5-point Likert scoring method.

In constructing the pool of items for the study, all duplicated items were identified and only one version was used. This removed 15 items from the initial 47 comprising the two existing scales. These were replaced by 12 new items created by the authors from the literature reviewed. This resulted in a pool of 44 items for use in the study.

Participants in the current study were asked to consider difficult times they have had in their life and to respond to the scale in relation to how they felt living through those difficult times by indicating on a 5-point Likert scale how much each item was true for them.

*Psychological Capital* was measured using the *Psychological Capital Questionnaire (PCQ-24)* which is a 24-item measure combining measures of hope, optimism, self-efficacy, and resilience in terms of the model developed by Luthans et al. (2007b). The scale has been used in a number of studies which have demonstrated its validity (Luthans et al. 2007b; Luthans, Avolio, Avey, & Norman, 2007a). All items were measured using a 6-point Likert scale of agreement with response options ranging from 1 – *strongly disagree* to 6 – *strongly agree*. Overall scale Cronbach's alpha in this study was .88 with individual subscale alphas of hope (*α* = .83), optimism (*α* = .82), self-efficacy (*α* = .85), and resilience (*α* = .80).

#### Life engagement

This was measured by a 6-item scale, the *life engagement test* (Scheier et al., 2006), designed to measure purpose in life, defined in terms of the extent to which a person engages in activities that are personally valued. In these data, it has an internal consistency of *α* = .86.

#### Self-determination

This was measured using a 6-item scale developed for the study (Appendix). The scale has a Cronbach's alpha of .85 and confirmatory factor analysis (CFA) fit statistics show the items are a good fit for the data (*χ* ^2^ (9) = 48.59; *p* < .001; Incremental Fit Index (IFI) = .97; Comparative Fit Index (CFI) = .97; root mean square error of approximation (RMSEA) = .06; standardised root mean square residual (SRMR) = .03).

#### Perceived social support

Perceived levels of social support received from family and friends were measured by the *Perceived Social Support Scale* (Procidino & Heller, 1983). This measure consists of two 20-item subscales addressing perceived social support from family members and friends, respectively. Most items appear on both subscales with identical wording, apart from changes in the referent of the statement (e.g. “Members of my family are good at helping me solve problems” vs. “My friends are good at helping me solve problems”). The measure is designed to reflect a variety of instances of support including emotional, information, feedback, and reciprocity (i.e. provision of support by the individual). In the current study, the Cronbach's alpha values were family support (*α* = .81) and support from friends (*α* = .83). This compares favourably with previous reported alpha values, for example, family support (*α* = .87) and support from friends (*α* = .88) (Dubois, Felner, Brand, Adan, & Evans, 1992). Predictive validity has been established in longitudinal studies with regard to a variety of measures of psychological distress (Dubois et al., 1992), and the scale has been shown to be correlated with a range of other relevant variables such as social competence (Procidano & Heller, 1983).

#### Student stressors

This was measured using the *Inventory of College Student's Recent Life Experiences* (ICSRLE) (Kohn, Lafreniere, & Gurevich, 1990, 1991; Kohn, O'Brien, & Pickering, 1997) which is a 49-item scale measuring the influence of everyday stressors on the physical and mental health of university students specifically. Items are rated on a 4-point Likert scale for the frequency of participants’ experiences with hassles over the past month, with 1 = not part of my life and 4 = very much part of my life. Sample items include: “being let down or disappointed by friends” and “not enough time to meet your obligations”. Internal consistency for the ICSRLE for the current sample was *α* = .89.

### Procedure

Convenience sampling was used to distribute an email embedded link to an online questionnaire to 1430 students. A total of 855 fully completed responses were received, at a response rate of 59.8%. Incomplete questionnaires were not processed.

### Statistical analysis

The maximum-likelihood method of factor analysis with oblique rotations was used to identify the factor structure of the item pool. Reliability analysis then tested the internal consistency of the factors. The analysis of moment structures (AMOS) programme was then used to run the CFA on the items, followed by the structural equation modelling (SEM) to test a model of the relationship between benefit finding and a range of other measures.

## Results

**Figure 1.**
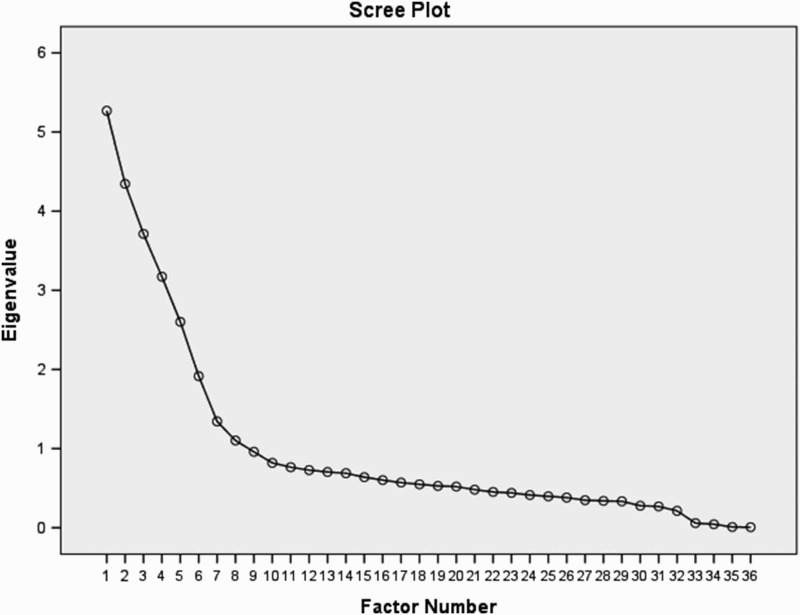
Scree plot from factor analysis.

The 44 items from the original scales were submitted to an exploratory factor analysis (EFA) with the maximum-likelihood extraction and oblique rotation (direct oblimin in statistical package for the social sciences [SPSS]) into a simple structure. The Kaiser–Meyer–Oklin value was .807 and Bartlett's Test of Sphericity reached significance (*χ* ^2 ^= 8400.14, df = 1035, *p* < .001). Thirteen factors were found with eigenvalues greater than 1 accounting for 61% of the variance; however, the scree plot suggests a maximum 6-factor solution (Figure 1). This was supported by a review of the items loading on the rotated factors which suggest that only 6 factors can be identified. All factors beyond these 6 have only 2 items loading on them. These 6 factors account for 43.2% of the variance. These factors with factor loadings and Cronbach's alphas are shown in Table 1. The factors were labelled, *acceptance (α = .86), family bonds (α = .76), personal growth (α = .81), relationships (α = .83), empathy (α = .80),* and *reprioritisation (α = .82).* This produced a 28-item *General Benefit Finding Scale* (GBFS)*,* consisting of 12 items from the Tomich and Helgeson scale, 8 items from the McMillen and Fisher scale, and 8 new items (as shown in Table 1).

**Table 1.** Items, factor loadings, and psychometric data for the new scale.

Total % variance = 43.2	Factor loadings
**Acceptance: *α* = .86, Eigenvalue = 4.19, % variance = 9.1**	
Led me to be more accepting of things	.78
Taught me how to adjust to things I cannot change	.75
Helped me take things as they come	.56
Given me a more realistic set of expectations	.54
Taught me to be patient	.48
**Family bonds: *α* = .76, Eigenvalue = 3.40, % variance = 7.4**	
Brought my family closer together	.68
Made me more sensitive to family issues	.65
Helped me appreciate my family more	.54
*Made me more aware of what my family means to me*	.44
**Growth: *α* = .81, Eigenvalue = 3.30, % variance = 7.2**	
Made me a more effective person	.76
*Taught me how to cope more effectively*	.69
Helped me become a stronger person	.67
*Taught me how I can handle most things*	.49
Led me to deal better with problems	.47
Helped me to grow emotionally and spiritually	.45
**Relationships: *α* = .83, Eigenvalue = 3.11, % variance = 6.8**	
Helped me become more aware of the support available from others	.75
Helped me realise who my real friends are	.66
Led me to feel more positive about others	.62
Led me to meet people who have become some of my best friends	.61
**Empathy: *α* = .80, Eigenvalue = 2.97, % variance = 6.5**	
*Made me more compassionate to those in similar situations*	.68
*Made me more sensitive to the needs of others*	.68
Made me care more about others	.54
*Made me closer to people I care about*	.53
Taught me that everyone has a right to be valued	.51
**Reprioritisation: *α* = .82, Eigenvalue = 2.84, % variance = 6.2**	
*Led me to place less emphasis on material things*	.86
*Led me to live my life more simply*	.79
Led me to change my priorities in life	.69
Helped me become more focused on real priorities	.51

Note: Underlined items come from the Tomich and Helgeson scale. Items in italics come from the McMillen and Fisher scale. Other items were created by the authors.

### Psychometric properties

We used the set of criteria proposed by Lampling et al. (2002) and summarised by Smith et al. (2005) to set out some of the psychometric properties of the data (Table 2). The data used in this study were based on a cross-sectional survey, so test–retest reliability was not tested. However, the items and factors perform well on all the other dimensions as shown in Table 2.

**Table 2.** Psychometric criteria and scale performance.

Property	Criteria for acceptability	Performance of scale
Item analysis/reduction	All items should have factor loadings > .30	Factor loadings range .44–.86
	Missing data < 5%	No missing data
	Inter-item correlations < .75	Inter-item correlations range .38–.71
	Item total correlations ≥ .25	Item total correlations range .46–.82
	Maximum endorsement frequency < 80%	Maximum endorsement frequency = 76.2%
	Minimum adjacent endorsement > 10%	Minimum adjacent endorsement = 14.6%
Acceptability	Skewness values < 1	Maximum skewness .96
	Missing data < 5%	No missing data
Reliability	Cronbach's alpha > .70	Cronbach's alphas’ range .76–.86
	Item total correlations ≥ .20	Item total correlations range .50–.83

The next stage was to run a CFA using the AMOS programme on SPSS. In testing model fit, there is still some debate about cut-off values for goodness of fit. For this study, we used the review by Hooper, Coughlan, and Mullan (2008), and specifically the following cut-offs. A non-significant chi-square or a chi-square to degrees of freedom ratio of 3:1 or less, a CFI of .95 or greater, an IFI of .95 or greater, a RMSEA of .08 or less (ideally as close to .05 as possible), and a SRMR of less than .08. The initial model had a *χ* ^2^ of 48.98 with nine degrees of freedom (*p* < .001), with a CFI = .98, an IFI = .98, a RMSEA = .08, and a SRMR = .03. The Modification Index indicated that the model fit could be improved by allowing *reprioritisation* to correlate with both *acceptance* and *relationships*, as shown in Figure 1. The new model thus produced had a *χ* ^2^ of 24.76 with nine degrees of freedom (*p* < .001), with a CFI = .99, an IFI = .99, a RMSEA = .05 (CI: .03–07), and SRMR = .03. Although the *χ* ^2^ is significant, this may be because of the large sample size, and the relative/normed *χ* ^2^ (*χ* ^2^:df) is 2.8:1 which is lower than the accepted cut-off of 3:1 recommended by Kline (2005). Overall the model is a good fit.

Although the EFA identified a 6-factor solution composed of items from both the Tomich and Helgeson (2004) and the McMillen and Fisher (1998) scales and failed to support either scale, we felt it useful to use confirmatory factor analysis (CFA) to test each scale separately in order to compare them with the new scale. The CFA of the Tomich and Helgeson (2004) scale produced a *χ* ^2^ of 45.93 with eight degrees of freedom (*p* < .001). The CFI =.88, the IFI =.88; the RMSEA = .15, and the SRMR =.19. The CFA of the Tomich and Helgeson (2004) scale produced a *χ* ^2^ of 95.80 with 11 degrees of freedom (*p* < .001). The CFI =.79, the IFI =.79, the RMSEA =.11, and the SRMR =.14. We concluded that the new model performed significantly better than any of the original models.

The next stage in analysis was to produce some descriptive statistics and correlations as shown in Table 3. Benefit finding and its component factors were all significantly positively correlated with the dimensions of psychological capital, social support, and student hassles, but no correlations were above .7 indicating that there was no problem with multicollinearity.

**Table 3.** Descriptive statistics and correlations.

		Mean (Sd)	1	2	3	4	5	6	7	8	9	10	11	12	13	14	15	16	17	18	19	20
1	Benefit finding	3.2(1.0)	1.0																			
2	Acceptance	3.3(1.2)	0.79																			
3	Family bonds	3.4(1.2)	0.84	0.59																		
4	Relationships	3.1(1.0)	0.81	0.62	0.60																	
5	Growth	3.1(1.1)	0.79	0.51	0.60	0.59																
6	Reprioritisation	3.0(1.2)	0.82	0.54	0.66	0.57	0.58															
7	Empathy	3.2(1.2)	0.86	0.60	0.64	0.66	0.62	0.68														
8	Resilience	3.0(1.1)	0.44	0.43	0.31	0.39	0.38	0.37	0.31													
9	Life engagement	3.1(1.1)	0.52	0.41	0.44	0.46	0.40	0.40	0.46	0.25												
10	Optimism	3.3(1.1)	0.62	0.50	0.51	0.51	0.51	0.50	0.51	0.33	0.40											
11	Self-determination	4.1(1.8)	0.50	0.39	0.37	0.46	0.41	0.42	0.40	0.33	0.32	0.41										
12	Self-efficacy	3.1(1.1)	0.35	0.33	0.30	0.26	0.22	0.28	0.32	0.22	0.24	0.28	0.26									
13	Hope	4.2(2.1)	0.48	0.38	0.44	0.41	0.33	0.38	0.45	0.21	0.40	0.31	0.29	0.37								
14	Friend support	2.9(1.3)	0.60	0.48	0.49	0.50	0.49	0.50	0.51	0.28	0.32	0.38	0.29	0.22	0.31							
15	Family support	2.9(1.4)	0.57	0.45	0.47	0.48	0.44	0.49	0.50	0.22	0.23	0.34	0.27	0.18	0.31	0.65						
16	Developmental challenge	4.9(2.5)	−0.47	−0.40	−0.32	−0.41	−0.40	−0.39	−0.37	−0.39	−0.16	−0.42	−0.28	−0.13	−0.17	−0.29	−0.23					
17	Time pressure	9.1(3.3)	−0.57	−0.50	−0.48	−0.50	−0.40	−0.41	−0.49	−0.32	−0.35	−0.47	−0.36	−0.32	−0.33	−0.38	−0.32	0.31				
18	Social issues	5.2(3.0)	−0.51	−0.39	−0.42	−0.45	−0.40	−0.45	−0.40	−0.27	−0.32	−0.37	−0.31	−0.22	−0.25	−0.34	−0.27	0.32	0.45			
19	Academic alienation	3.0(1.7)	−0.34	−0.28	−0.27	−0.36	−0.24	−0.28	−0.23	−0.26	−0.24	−0.27	−0.18	−0.13	−0.22	−0.25	−0.16	0.20	0.21	0.36		
20	Romantic problems	3.2(1.5)	−0.41	−0.35	−0.34	−0.40	−0.33	−0.31	−0.31	−0.40	−0.25	−0.29	−0.27	−0.13	−0.16	−0.28	−0.20	0.31	0.25	0.32	0.26	
21	Friend problems	3.5(1.6)	−0.56	−0.45	−0.46	−0.51	−0.45	−0.42	−0.44	−0.23	−0.36	−0.34	−0.32	−0.20	−0.30	−0.32	−0.31	0.27	0.42	0.38	0.28	0.28


Note: All correlations significant at *p* < .001.

**Figure 2.**
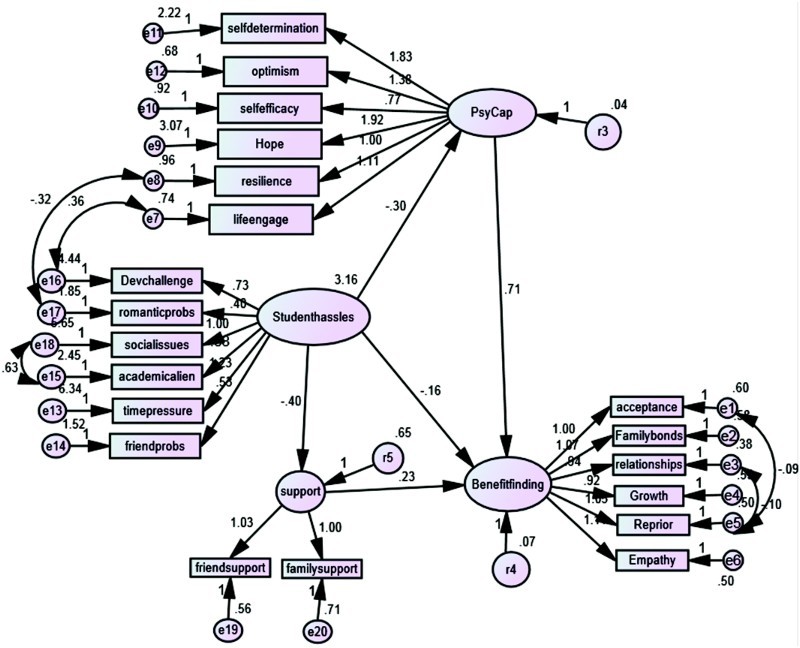
Structural equation model for benefit finding on psychological capital, social support and student daily hassles. Chi-square (162) = 419.83, *p* < .001; IFI = .95; CFI = .95; RMSEA = −.05 (CI = .046−.056).

The next stage was to test the proposed model using SEM with AMOS as shown in Figure 2. The model was a good fit for the data (*χ* ^2^ (162) = 419.83; *p* < .001; IFI = .95; CFI = .95; RMSEA = .05). Although the *χ* ^2^ is significant, this may be because of the large sample size and the relative/normed *χ* ^2^ (*χ* ^2^:df) is 2.6:1 which is lower than the accepted cut-off of 3:1 recommended by Kline (2005). Bootstrapping was used with 1000 samples and the standardised indirect effect of student hassle on benefit finding was −.594, *p* < .001 (95% CL: −.845 to −.359).

In order to identify mediation effects, the standardised regression coefficients from the SEM analysis were extrapolated and are shown in Table 4. The regression coefficients allow a comparison of the direct effects of the predictor variable (student hassles) on the outcome variable (benefit finding) with and without the mediator variable(s). If the mediator variable has no effect, the regression coefficients for student hassles on benefit finding should remain the same with or without the mediator added. If mediation is occurring, the coefficient with the mediator added should be lower than the coefficient without the mediator. If the coefficient with the mediator is reduced to non-significance, we can say that full mediation has occurred. From Table 4, we can see that the effect with the mediator(s) added was lower but still significant. Hence, we can conclude that psychological capital and social support separately provided partial mediation of the effect of student hassles, and when both psychological capital and social resources were included their combined effect was larger but still partial mediation.

**Table 4.** Mediation analysis based on standardised regression weights from SEM.

Relationship	Mediator	Direct effect without mediator	Direct effect with mediator	Indirect effect	Mediation
Student hassles with benefit finding	Social support	−.456***	−.361***	−.192**	Partial
	Psychological capital	−.456***	−.224***	−.481***	Partial
	Social support and Psychological capital	−.456***	−.173***	−.594***	Partial

**p < .01.

***p < .001.

## Discussion

The purpose of the current study was, (1) to develop a general measure of benefit finding which could be used outside of the field of chronic illness, disability, and severe trauma and (2) to test a model whereby psychological and social resources mediate the relationship between stressors and benefit finding. The 28-item GBFS with its six dimensions herein produced demonstrates good internal reliability, and promising psychometric properties. Drawing on the two well-established scales (McMillen & Fisher, 1998; Tomich & Helgeson, 2004), it can be argued that the GBFS covers the domain of benefit finding previously defined in the literature. Each factor is clearly supported by good levels of internal consistency and the criteria proposed by Lampling et al. (2002) and summarised by Smith et al. (2005) are clearly met. In addition, all six factors of the GBFS correlate significantly positively with psychological capital and social support and significantly negatively with the measure of student hassles. This provides some evidence of concurrent validity. Confirmatory factor analysis also shows this six-factor model to be an acceptable fit for the data.

While one purpose of the study was to develop the measure, the second was to test the proposed model and the SEM analysis reported shows that the model is indeed a good fit for the data. The model confirms a number of suggestions from the introduction. Firstly, it confirms the extended model of psychological capital proposed with the additional variables of self-determination and life engagement. While the original four-dimension model proposed by Luthans et al. (2007b) with the four dimensions of optimism, hope, self-efficacy, and resilience was a reasonable fit the six-dimension model with the addition of self-determination and life engagement was a very good fit. This suggests that the inclusion of life engagement and self-determination improves the explanatory power of the model substantially. Secondly, the negative relationship between student hassles and benefit finding supports some emerging evidence (Cassidy, 2012) that the role of benefit finding in relation to everyday levels of stress may be different from that shown in relation to trauma or chronic illness and may be more akin to the experience of care givers. This is a somewhat controversial point as it would appear to contradict previous research which assumes that threat severity increases benefit finding (Helgeson et al., 2006; Park et al., 1996). However, there are a number of points to be made. Firstly, the majority of evidence thus far has come from research on trauma survivors or patients with chronic illness (Helgeson et al., 2006). Arguably, an important difference compared with more general life stress is that the events experienced have been life threatening. In a recent meta-analysis, Shakespeare-Finch and Lurie-Beck (2013) found both a linear and a curvilinear relationship between symptoms of post traumatic stress disorder (PTSD) and post-traumatic growth. The curvilinear relationship was stronger but the linearity of the relationship seems to depend on the type of event and age of individual among other things. This along with some other emerging evidence (Kleim & Ehlers, 2009) would suggest that the relationship between stress and growth or benefit finding is not as consistent or simple as previously suggested. Secondly, traumatic events and chronic illness cannot really be compared with everyday stressors in terms of their potential stress load. Given that their stress load is likely to start at a relatively high stress level, their measurement cannot be on the same continuum from low to high that would be used to evaluate everyday stressors. Finally, the current study used a measure of perceived stress in a cross-sectional study and the relationship between the measure and benefit finding may be reciprocal rather than unidirectional.

Given that benefit finding has been previously shown to predict a range of positive health outcomes, the current data contribute to our understanding of the context within which benefit may be found in response to general life stress. On the one hand, it supports the extant literature on the role of psychological and social resources in the psychology of the person, and perhaps serves as a warning not to throw the baby out with the bath water in relation to the traditional stress literature. On the other hand, it fits with the growing positive psychology literature which shows that a focus on what works may be a useful and informative addition to what we learn from studying what is broken. In other words, studying healthy people may be more productive than just studying those who are ill in developing strategies to prevent illness and promote positive health. There are a growing number of “resilience building” interventions such as, the Penn Resiliency Project (Cardemil, Reivich, Beevers, Seligman, & James, 2007; Chaplin et al., 2006; Cutuli, Chaplin, Gillham, Reivich, & Seligman, 2006), the International Resilience Project (Ungar, 2011a, 2011b; Ungar, Duque, & Hernandez, 2011; Ungar & Liebenberg, 2011), and the Young Foundation Programme (Daniel & Wassell, 2002; Gilligan, 2009; Morris, 2009). The argument from this study is that they need to focus across the range of psychological and social resources if they are to be successful.

The cross-sectional nature of this study and its student sample are limitations; however, there is good reason, as outlined above, to suggest that the GBFS is a reliable measure, fit for purpose in exploring the mechanisms linking benefit finding to health within a stress-health model. In terms of the benefit finding model clearly longitudinal data from a wider range of the population would allow more confident decisions to be drawn. On the other hand, the findings do fit with some previous research and make a worthwhile contribution to the growing literature. The study also focuses on positive change which may be a limitation and in no way suggests that the authors advocate a neglect of negative changes. Both are important in gaining a complete understanding. Furthermore, the measures used here assess perceived benefit finding which may be subject to positive bias in reporting. However, it is justifiably arguable that it is the individual's construction of the event which impacts on their well-being.

## Conclusion

The current study suggests that benefit finding is more likely to develop in the context of psychological and social resources and is inversely related to the level of stress experienced. The implications of this are that we may begin to understand the context within which benefit finding is likely to occur and thus provides a focus for potential intervention. There is a growing field of positive psychology-based resilience building interventions which could be used successfully. The findings support the established literature which attests to the importance of social support and related interventions (e.g. support groups). The role of psychological capital provides further direction for innovative interventions which can build its component parts, resilience, hope, optimism, and self-efficacy, and can extend to life engagement and self-determination. The measure and the model proposed herein can be used to extend the evidence base and to guide and evaluate interventions.

## Appendix. Self-determination scale

**Table:**

Self-determination scale					
Thinking about the things you do in your daily life, please rate how often the following statements are true of you	Never	Seldom	Sometimes	Often	Always
1. I make my own hoices in life	0	1	2	3	4
2. I enjoy the things I do	0	1	2	3	4
3. I do things because I enjoy them	0	1	2	3	4
4. The things I do satisfy me	0	1	2	3	4
5. I get a sense of self-worth from the things I do	0	1	2	3	4
6. I make my own decisions in life	0	1	2	3	4
